# FAK Deficiency in Bone Marrow Stromal Cells Alters Their Homeostasis and Drives Abnormal Proliferation and Differentiation of Haematopoietic Stem Cells

**DOI:** 10.3390/cells9030646

**Published:** 2020-03-06

**Authors:** Yuenv Wu, Lydia Campos, Elisabeth Daguenet, Zhiguo He, Tiphanie Picot, Emmanuelle Tavernier-Tardy, Gilbert Soglu, Denis Guyotat, Carmen-Mariana Aanei

**Affiliations:** 1 Laboratoire d’Hématologie, CHU de Saint-Etienne, 42055 Saint-Etienne CEDEX, France; wynalexa@163.com (Y.W.); lydia.campos@chu-st-etienne.fr (L.C.); Tiphanie.Picot@chu-st-etienne.fr (T.P.); 2 UMR 5239, Laboratoire de Biologie et Modélisation de la Cellule, 69364 Lyon, France; emmanuelle.tavernier@icloire.fr (E.T.-T.); denis.guyotat@icloire.fr (D.G.); 3 Département d’Hématologie, Institut de Cancérologie Lucien Neuwirth, 42270 Saint-Priest-en-Jarez CEDEX, France; Elisabeth.DAGUENET@icloire.fr (E.D.); Gilbert.SOGLU@icloire.fr (G.S.); 4 Biologie, Ingénierie et Imagerie de la Greffe de Cornée (BiiGC), Université Jean Monnet, 42270 Saint-Priest-en-Jarez, France; hezhiguo@hotmail.fr

**Keywords:** bone marrow stromal cells (BMSCs), focal adhesion kinase (FAK), myelodysplastic syndromes (MDS), haematopoietic stem precursor cell (HSPC)–BMSC interaction, adhesion molecules, lymphocyte function-associated antigen 1 (LFA-1), CD44

## Abstract

Emerging evidence indicates that in myelodysplastic syndromes (MDS), the bone marrow (BM) microenvironment may also contribute to the ineffective, malignant haematopoiesis in addition to the intrinsic abnormalities of haematopoietic stem precursor cells (HSPCs). The BM microenvironment influences malignant haematopoiesis through indirect mechanisms, but the processes by which the BM microenvironment directly contributes to MDS initiation and progression have not yet been elucidated. Our previous data showed that BM-derived stromal cells (BMSCs) from MDS patients have an abnormal expression of focal adhesion kinase (FAK). In this study, we characterise the morpho-phenotypic features and the functional alterations of BMSCs from MDS patients and in FAK knock-downed HS-5 cells. The decreased expression of FAK or its phosphorylated form in BMSCs from low-risk (LR) MDS directly correlates with BMSCs’ functional deficiency and is associated with a reduced level of haemoglobin. The downregulation of FAK in HS-5 cells alters their morphology, proliferation, and differentiation capabilities and impairs the expression of several adhesion molecules. In addition, we examine the CD34+ healthy donor (HD)-derived HSPCs’ properties when co-cultured with FAK-deficient BMSCs. Both abnormal proliferation and the impaired erythroid differentiation capacity of HD-HSPCs were observed. Together, these results demonstrate that stromal adhesion mechanisms mediated by FAK are crucial for regulating HSPCs’ homeostasis.

## 1. Introduction

Myelodysplastic syndromes (MDS) are considered a heterogeneous group of clonal haematopoietic disorders in which spliceosome mutations cooperate with specific epigenetic modifiers in HSPCs to raise the MDS phenotype. Current therapies, which are designed to target HSPC malignant clones, have limited efficacy: They slow the evolution towards acute myeloid leukaemia (AML) rather than stopping clonal evolution and eradicating the disease. In the last few years, the contribution of the microenvironment in MDS pathogenesis has largely been accepted. However, the mechanism by which MDS arises remains unknown, and it is not clear whether the intrinsic abnormalities of haematopoietic stem precursor cells (HSPCs) or the bone marrow (BM) microenvironment trigger the initial pathological process.

In support of the ‘HSPC-first’ concept, Meydouf et al. have shown that MDS HSPCs reprogram the bone marrow stromal cells (BMSCs) to favour MDS haematopoiesis [1]. Runx2 downregulation in HSPCs impairs osteogenic differentiation of bone marrow stromal cells (BMSCs) in MDS through the Notch-Hes pathway [2].

More recently, two main concepts regarding the role of the BM microenvironment in MDS pathogenesis have emerged: ‘The niche-induced malignant transformation’ and the ‘niche-facilitated malignant transformation’ [3].

Regarding the latter, it has been shown that the BM inflammatory microenvironment plays a major role in MDS pathogenesis. The deletion of the Shwachman Bodian–Diamond-Syndrome (SBDS) gene (coding for ribosome maturation protein SBDS) results in P53 activation in the BMSCs, with the activation of downstream transcriptional pathways and the release of alarmins [4]. The priming of the BM microenvironment by alarmins (S100A8/A9) then triggers inflammatory alterations in the BM niche via the nucleotide-binding and oligomerization domain (NOD)-like receptor protein 3 (NLRP3), thus contributing to the onset of the lytic form of cell death—called pyroptosis—and β-catenin activation [5,6]. These processes cause genotoxic stress in HSPCs with the expression of splicing variants through a TP53-S100A8A9-TLR signalling cascade [4]. In addition, the MSCs isolated from patients with SBDS are unable to support angiogenesis, and they also contribute to haematopoietic dysfunction [7].

Similarly, the BM microenvironment is responsible for disrupting immunosurveillance, thereby allowing for the expansion of abnormal myeloid progenitors and the accumulation of cells with DNA damage [8]. Moreover, BMSCs exert substantial immunosuppressive activity by paracrine signals, cell-to-cell interaction, and direct T-cell inhibition through the synthesis of indoleamine 2,3-dioxygenase (IDO) [9].

The BM microenvironment contributes to the malignant transformation of HSPCs through the disruption of specific signalling pathways (e.g., p38 MAPK [9], β-catenin (Ctnnb1)/WNT [10,11,12]) and the perturbation of the epigenetic regulatory system (e.g., abnormal methylation of human Hh-interacting protein (HHIP) in AML/MDS-derived BMSCs [13]). The reduced expression of FRZB (SFRP3) in BMSCs via epigenetic silencing and the consequent activation of the Wnt/β-catenin pathway in HSPCs may also contribute to the disease progression of MDS [14]. However, not all of these results provide sufficient evidence to sustain the hypothesis that the BM microenvironment induces the malignant transformation of HSPCs.

The direct contact between haematopoietic stem cells (HSCs) and BMSCs through adhesion receptors and their ligands is highly important for HSCs’ retention in the BM niche [15] as a prerequisite for proper HSC function [16].

However, the role of adhesion-mediated processes between these cells in the BM niche and their contribution to the pathogenic processes in myeloid malignancies remain largely unknown. Our previous data [17,18] demonstrated that BMSCs from MDS patients display an abnormal expression of focal adhesion kinase (FAK), an intracellular master regulator of cell adhesion [19,20]. FAK is a cytoplasmic tyrosine kinase that, once activated, initiates a signalling cascade through PI3K-Akt and MAPK pathways, which in turn promotes cell survival, cell growth, mobility, and angiogenesis [21].

In the present study, we confirm the pathological role of BMSCs in MDS settings. To further demonstrate the function of FAK in this process, we have developed an in vitro system that allows us to assess whether the anomalies observed in BMSCs are related to abnormal FAK expression. FAK knockdown in the HS-5 human stroma cell line using short hairpin RNA (shRNA) recapitulates the morphological and functional features as well as the abnormal expression of adhesion molecules observed in BMSCs from low-risk MDS (LR-MDS) patients. To determine whether adhesion-related mechanisms occur in LR-MDS settings, we evaluated the impact of FAK downregulation in HS-5 cells on CD34+ healthy donors’ haematopoietic stem cells (HD-HSCs) after direct and indirect in vitro co-cultures. We showed that FAK in BMSCs is an important regulator of HSPCs’ homeostasis. A deficiency of FAK in BMSCs favours the initial expansion of immature CD34+ HD-HSPCs with their exhaustion in long-term co-cultures. In addition, an abnormal differentiation of HSPCs, especially towards erythroid lineage, was observed after direct contact with FAK-deficient BMSCs. These effects were significantly correlated with the alteration of several signalisation pathways. Rescuing FAK expression, even partially, restores the expression of the signalling proteins involved in BMSCs’ homeostasis.

Taken together, these results support the hypothesis that FAK expression in BMSCs is required for normal HSPC proliferation and differentiation. Therefore, in the context of LR-MDS, restoring the expression of FAK in BMSCs could improve cytopenia, especially anaemia, and could be further exploited to improve MDS patient care.

## 2. Materials and Methods 

### 2.1. Bone Marrow Stromal Cells (BMSCs) and HS-5 Culture Setting

#### 2.1.1. Setting of Bone Marrow Stromal Cells (BMSC) Primary Cultures from Myelodysplastic Syndromes (MDS) Patients

BM samples were obtained from patients diagnosed with MDS and from healthy donors (HD) after obtaining written informed consent, as approved by the institutional procedures of the independent ethics committee and the ‘Comité de Protection des Personnes’-Ile de France (NCT03233074/17.07.2017). HDs were aged matched and had blood counts in the normal range. Detailed characteristics of MDS patients are shown in Table 1.

BM mononuclear cells (BMMNCs) were isolated using Lymphosep (Biowest, Nuaillé, France) gradient separation. 1 × 10^6^ cells were cultured in T25 cell flasks in MesenCult® MSC basal medium (Stem Cell Technologies, Vancouver, BC, Canada) and Mesenchymal Stem Cell Stimulatory Supplement (Stem Cell Technologies, Vancouver, BC, Canada) and in 1% penicillin/streptomycin (Lonza, Basel, Switzerland) at 37 °C in 5% CO_2_, with medium replacement twice a week in the beginning of the cultures and every week after date until 80% of confluence is reached.

The resulting CD45-CD73+ CD105+ CD90+ (purity >98%), low-passage BM-stromal cells were used to perform different tests.

Adherent cells were analysed by flow cytometry. The list of antibodies used for BMSC discrimination and characterisation is shown in Table 2.

#### 2.1.2. Setting HS-5 Cell Line Cultures 

The human HS-5 stromal cell line (ATCC, Manassas, VA, USA) was cultured in low-glucose Dulbecco’s Modified Eagle’s medium (ATCC) supplemented with 10% Fetal Calf Serum (FCS; Gibco by Thermo Fisher Scientific, Illkirch Cedex, France), 4mM L-glutamine (Lonza) and 1% penicillin/streptomycin (Lonza, Basel, Switzerland) at 37 °C in 5% CO_2_ during a maximum of 18 passages [22]. Cell cultures were assessed under an inverted microscope IX81 (Olympus, Tokyo, Japan).

### 2.2. Optical Microscopy

BM-derived MSCs and HS-5 cells were fixed with methanol for 10 min at room temperature (RT). After drying, cells were stained with Giemsa solution (Sigma Aldrich, St. Quentin Fallavier Cedex, France) for 45 min at RT and evaluated under optical microscopy (Zeiss, Berlin, Germany).

### 2.3. Cell Growth and Cell Cycle Evaluation in Bone Marrow Stromal Cell (BMSC) and HS-5 Cells 

#### 2.3.1. Proliferation Assays

The proliferation of MSCs and HS-5 cells was assessed at days 7 and 14 of cultures using the MST assay (CellTiter 96® AQueous, Promega, France). Cells were seeded in triplicate in 96-well plates at 10^3^ cells/well density. 20μL of CellTiter 96® AQueous solution was added to each well before recording an absorbance reading at 490nm.

Alternatively, the proliferation rate of HS-5 cells was evaluated by carboxyfluorescein-diacetate-succinimidyl-ester staining (BD Horizon CFSE, BD Bioscience, Le Pont de Claix Cedex, France), as previously described [23]. The population doublings (PD) were calculated according to the following formula: PD = log_10_ (N_h_/N_i_) × 3.33, where N_h_ and N_i_ are the numbers of harvested and initially plated cells, respectively.

#### 2.3.2. Cell Cycle Evaluation

Hoechst 33342 (BD Bioscience, Le Pont de Claix Cedex, France) and Pyronin Y (Sigma-Aldrich, St. Quentin Fallavier Cedex, France) were used for cell cycle analysis. 1×10^6^ cells were fixed overnight in ice-cold ethanol [70% (*v/v*) in water] at −20 °C. Fixed and permeabilised cells with BD Cytofix/Cytoperm Buffer (BD Biosciences) were stained with 2 μg/mL Hoechst 33342 and 4 μg/mL Pyronin Y (Sigma-Aldrich). 10^5^ events were collected from each sample using an FACS Canto II flow cytometer. Cell cycle analysis was performed using FlowJo software (TreeStar Inc., Ashland, OR, USA).

### 2.4. Colony Forming Unit-Fibroblast Assay

10^6^ BMMNCs were seeded in 35 mm dishes in duplicate. Cells were incubated for 10 days; they were then fixed with 4% paraformaldehyde (PFA) and stained with 0.1% toluidine blue (Sigma-Aldrich, St. Quentin Fallavier Cedex, France) for 60 min at RT. Colonies (defined as consisting of at least 30 cells) were counted under a light microscope (200× magnification). The size of the colonies was measured using Image J 1.51s software (NIH, Bethesda, MD, USA).

### 2.5. In vitro Adipogenic and Osteogenic Differentiation of Bone Marrow Stromal Cells (BMSCs) Derived from Healthy Donors (HD) and Myelodysplastic Syndromes (MDS) BM Aspirates and Cytochemical Staining

For adipogenic differentiation, cells were cultured in AdipoDiff medium (Miltenyi Biotec, Bergisch Gladbach, Germany) supplemented with 1% penicillin/streptomycin (Lonza, Basel, Switzerland). Thereafter, cells were fixed with 4% paraformaldehyde (PFA) for 15 min and stained with Oil red O solution (Sigma-Aldrich, St. Quentin Fallavier Cedex, France) for 10 min, followed by two washes with distilled water. Cells with red lipid vacuoles were counted under a light microscope.

For osteogenic differentiation, cells were cultured in an OsteoDiff medium (Miltenyi Biotec, Bergisch Gladbach, Germany) supplemented with 1% penicillin/streptomycin (Lonza, Basel, Switzerland) and replaced every three days. Alizarin red staining (Sigma-Aldrich, St. Quentin Fallavier Cedex, France) was performed to analyse calcium deposits after cell fixation with 4% PFA for 15 min. Cells were washed with distilled water and stained with Alizarin red solution (Sigma-Aldrich, St. Quentin Fallavier Cedex, France) 40 mM, pH 4.2 for 10 min.

The calcium deposits were assessed under optical microscopy, and staining intensity was graded as follows: 0 = absent, 1 = 20%, 2 = 40%, 3 = 60%, 4 = 80%, 5 = 100%. Each condition was assayed in triplicate.

### 2.6. Wound-Healing Assay

To analyse spreading and invasiveness, 5 × 10^5^ HS-5 cells were seeded in 6-well culture plates for 24 h, after which a scratch with a 1000 μL pipette tip was made on the monolayer. Debris was removed by two washes with PBS. Next, cells were incubated in a complete growth medium at 37 °C, 5% CO_2_ in a humidified atmosphere. The invasiveness of the scratch was evaluated 24 h later under optical microscope (Zeiss microscope, Berlin, Germany, at 200× magnification). Image analysis was performed with Image J 1.51s software (NIH, Bethesda, MD, USA).

### 2.7. Focal Adhesion Kinase (FAK) Inhibition in HS-5 Cells Using an ATP-Competitive FAK Kinase Inhibitor, VS-4718

HS-5 FAK-WT cells were cultured in Dulbecco’s Modified Eagle’s Medium-low glucose (ATCC, Molsheim Cedex, France) supplemented with 10% FCS (Gibco) and 4mM L-glutamine (Lonza). Cells were seeded in 6-well plates at a density of 1 × 10^5^ per well. VS-4718 (CliniSciences, Nanterre, France) was dissolved in DMSO to a 10 mM final concentration, aliquoted and stored at −80 °C.

The concentration of 2 µM was chosen for the experiments after conducting a dose–response experiment using concentrations of 0.01, 0.1, 0.2, 0.5, 1, 2, 5, and 10 µM. 

### 2.8. ShRNA-Mediated Focal Adhesion Kinase (FAK) Downregulation and FAK Re-Expression in HS-5 Cells

ShRNA vectors and the production of viral particles were done based on the MISSION shRNA strategy from Sigma Aldrich, as previously described [24]. 1 × 10^5^ HS-5 cells cultured in T25 flasks in Dulbecco’s Modified Eagle’s Medium-low glucose (ATCC, Molsheim Cedex, France), supplemented with 10% FCS (Gibco) and 4 mM L-glutamine (Lonza), were infected with shRNA-containing viral particles targeting FAK or SHC001-Control shRNA at 5 MOI (Multiplicity of Infection, calculated as PFU/cell numbers) in a humidified atmosphere of 5% CO_2_ at 37 °C during 24 h. The medium was removed and replaced with fresh medium every three days. The selection of HS-5 cells displaying stable shRNA-FAK was performed with 5 µg/mL Puromycin (Sigma).

For FAK rescue experiments, HS-5 cells with stable FAK knock-down were transfected using a lentivirus containing wild-type full-length FAK.

### 2.9. Western Blotting

Equal amount of proteins from each sample were loaded and run on SDS-PAGE gels (BioRad, Philadelphia, PA, USA) and then transferred to the methanol-activated Hybond P 0.45 PVDF membranes (GE Healthcare Biosciences, Pittsburgh, PA, USA). Membrane blocking was performed with a TBS-T buffer containing 5% skimmed milk and followed by incubation with rabbit anti-FAK (Cell Signalling Technology, Danvers, MA, USA), rabbit anti-P21 (Cell Signalling Technology), rabbit anti-P16 (Cell Signalling Technology), rabbit anti-pFAK (Y397 Thermo Fisher, Waltham, MA, USA), mouse anti-Akt (BD Bioscience, San Jose, CA, USA), mouse anti-pAkt (S473 BD Bioscience, San Jose, CA, USA), rabbit anti-β-Actin (Cell Signalling Technology), and rabbit anti-α-Tubulin (Cell Signalling Technology) antibodies at 1:1000 dilution for 2 h at RT. After washing, antigen–antibody reactions were developed using anti-mouse or anti-rabbit Horseradish Peroxidase (HRP)-conjugated secondary antibodies (DAKO, Glostrup, Denmark) for 60 min at RT. Finally, the protein bands were visualised using an electrogenerated chemiluminescence (ECL) detection system. The amount of target protein was normalised to the structural protein (β-Actin or α-Tubulin). Adobe Photoshop CC version 2017.0.020161012.r.53X64 was used to compare the intensity of protein bands.

### 2.10. Quantitative Reverse Transcriptase-Polymerase Chain Reaction (qRT-PCR) 

The total ribonucleic acid (RNA) from HS-5 cells, HS-5 cells treated with VS-4718, HS-5 cells transfected with FAK-specific shRNA and control shRNA were extracted using TRIZOL (Invitrogen, Camarillo, CA, USA). For each sample, 1 µg of total RNA was reversely transcribed into complementary DNA (cDNA) using the M-MLV Reverse Transcriptase kit (Invitrogen). Primers were obtained from Applied Biosystems (Foster City, CA; Table 3). The GAPDH gene was used as an endogenous control to normalise expression data. Gene expression was detected by the SYBR Green method in the 7900HT Fast Real-Time PCR System (Applied Biosystems, Carlsbad, CA, USA). All real-time PCR assays were run using the following program: 50 °C for 2 min, 95 °C for 10 min, followed by 40 cycles at 95 °C for 15 s and 60 °C for 1 min. Relative gene expression was calculated using the 2-ΔΔct method after normalisation to the reference gene GAPDH.

### 2.11. Flow Cytometric Characterisation and Functional Assessment of Healthy Donors (HD) and Myelodyplastic Syndromes-Haematopoietic Stem Precursor Cells (MDS-HSPCs)

#### 2.11.1. Antigen Expression and Quantitative Analysis of Cell Subsets

After a washing in phosphate-buffered saline (PBS) containing 0.5% bovine serum albumin (BSA) and 0.09% azide (AZ; wash solution, WS), cells were resuspended in 100 μL WS. 1 × 10^6^ cells were incubated with an adequate concentration of antibodies (Table 4) for 15 min at RT in the dark. Samples were acquired on an FACSCanto II cytometer (Beckton Dickinson Biosciences, San Jose, CA, USA) equipped with FACSDiva software version 1.7 (BD Biosciences). Analyses were performed using Infinicyt software version 2.0 (Cytognos, Salamanca, Spain). Annexin V and 7-AAD staining (BD Bioscience, USA) were used to exclude nonviable cells. Unstained cells were used to assess background fluorescence. The results were expressed as the absolute cell count (dual-platform method). Experiments were performed in triplicate.

#### 2.11.2. Haematopoietic Stem Precursor Cells (HSPCs) Clonogenic Assay

Haematopoietic progenitor colony formation was determined by clonogenic assays in methylcellulose, as previously described [17,24].

#### 2.11.3. CD34+ HD-HSPCs CFSE Staining

CD34+ HD-HSPCs were labelled with BD Horizon CFSE (BD Biosciences) prior to a co-culture with HS-5 cells. 10^7^ HSPCs were stained with 1mL of pre-warmed (37 °C) CFSE-working solution (10 µM) and incubated for 10 min at 37 °C.

### 2.12. Co-Cultures of Purified CD34+ HD-HSPCs with Transfected HS-5 Cells

#### 2.12.1. Direct Co-Cultures 

5 × 10^5^ HS-5 cells transfected with FAK-specific shRNA or control shRNA cells were seeded in the 6-well plates for 24 h until cell attachment was achieved. CD34+ HD-HSPCs were enriched from the BMMNCs after CD34-PE staining (BD Bioscience) by using immune-magnetic separation (EasySep PE selection kit) according to the manufacturer’s protocol. 1 × 10^5^ CFSE-stained CD34+ HD-HSPCs were added to each well. The co-cultures were performed in MyeloCult™ H5100 (StemCell Technologies, Grenoble, France) supplemented with 10^−6^ M hydrocortisone (StemCell Technologies) for 14 days. The half medium was changed at day 7. At days 5 and 14, both cells types were collected and counted with Trypan Blue Solution, 0.4%. Next, the HS-5 cells and CD34+ HD-HSPCs were evaluated by flow cytometry for viability (7-AAD staining), proportion of cell subpopulations, and HSPC differentiation. The antibodies used for HSPC staining are listed in Table 4. Data analysis was performed using Infinicyt software version 2.0 (Cytognos).

#### 2.12.2. Indirect Co-Culture Condition

Indirect co-cultures of shFAK KD HS-5 and control shRNA cells with CD34+ HD-HSPCs were prepared in 6-well plates, where haematopoietic cells were separated from the stromal cells by a polycarbonate transmembrane filter in a Transwell filter system (pore size 0.4 μm; BD Falcon, Bedford, MA, USA). HS-5 cells were seeded in the lower chamber (1 × 10^4^ cells/cm^2^), and after four days, 1 × 10^5^ CFSE-stained CD34+ HD-HSPCs were added to the upper well. Cells were co-cultured for an additional 14 days.

### 2.13. Statistical Analysis

Data are presented as the mean and standard error of the mean (SEM). Statistical comparisons were performed via a two-tailed t-test using Prism 5.0c (GraphPad Software, La Jolla, CA, USA) or Microsoft Excel (Microsoft Office 2010 Software, USA).

Values of *p* < 0.05(*); *p* < 0.01(**); *p* < 0.001(***); *p* < 0.0001(****) were considered statistically significant differences.

## 3. Results

### 3.1. Focal Adhesion Kinase (FAK) Deficiency in Bone Marrow Stromal Cells Derived from Patients with Myelodysplastic Syndromes (MDS BMSCs) Impairs Their Normal Function and Correlates with Ineffective Haematopoiesis 

**Figure 1:**
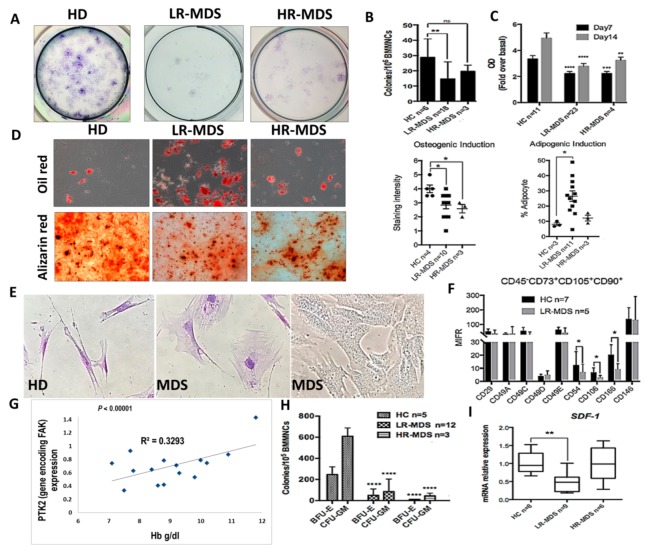
Intrinsic abnormalities related to focal adhesion kinase (FAK) deficiency in BMSCs from MDS patients correlate with the reduced clonogenic potential of HSPCs and with a degree of anaemia. (**A**,**B**) Evaluation of CFU-F and **C**, proliferative capacities (measured by MTT Cell Proliferation Assay) in BMSCs derived from MDS patients compared with healthy donors as controls (HC). (**D**) Quantification of oil red (adipogenic differentiation) and alizarin red (osteogenic differentiation) staining at day 14 in MSC derived from HC, LR-MDS (low-risk) and HR-MDS (high-risk) patients. (**E**) Morphological evaluation of MDS-derived MSCs compared to HC MSCs. (**F**) Phenotypic differences in BMSCs selected from LR-MDS patients compared to HC. (**G**) Significant correlation between PTK2 expression in BMSCs and the haemoglobin level in an MDS setting. (**H**) Evaluation of the clonogenic capacity of HSPCs selected from MDS patients compared to HC. I, SDF-1 mRNA expression in BMSCs isolated from LR-MDS and HR-MDS patients compared to HC. HC, HD controls; LR-MDS, low-risk MDS; HR-MDS, high-risk MDS. *p* < 0.05(*); *p* < 0.01(**); *p* < 0.0001(****).

We have previously reported that, in MDS stromal cells, the expression of total FAK and its phosphorylation at Tyr397 site were abnormal [17,18] and induced abnormal proliferation and differentiation with an increased propensity towards adipocyte differentiation to the detriment of osteogenesis [18]. We have also observed the gradual augmentation of FAK expression and activation during MDS progression [18]. Here, we show that, along with abnormal functional capacities (i.e., decreased proliferative and clonogenic capacities, increased propensity towards adipogenic differentiation, and reduced osteogenic differentiation (Figure 1A–D)), the abnormal expression of FAK in MDS-derived MSCs is associated with morphological and phenotypic changes (Figure 1E,F).

Large, flat, and granular stromal cells were observed in primary cultures of BMSCs from MDS patients compared with spindle-shaped cells in cultures from HD BM aspirates. Among the phenotypic changes, we observed that the BMSCs deficient in FAK from LR-MDS showed a diminution of expression of the CD106 immunomodulatory molecule, the CD166 osteogenic-related marker, and the CD54 (ICAM-1) adhesion molecules (Figure 1F).

A common biological characteristic of LR-MDS patients is anaemia. There was a strong positive correlation between the haemoglobin level and the level of PTK2 expression in BMSCs from LR-MDS (Figure 1G). In addition, the clonogenic capacities of HSPCs isolated from LR-MDS patients were significantly reduced (Figure 1H). Moreover, SDF-1 expression, an important cytokine for cell trafficking and the homing of CD34+ HSCs, was decreased in LR-MDS BMSCs (Figure 1I).

Thus, these data support the idea that FAK-deficient stroma might contribute to the MDS pathogenesis through abnormal differentiation and the capacity to generate osteoblasts, together with a reduced expression of several haematopoiesis-supporting molecules. 

### 3.2. The Inhibition of Focal Adhesion Kinase (FAK) Phosphorylation or FAK Expression in the HS-5 Cell Line Recapitulates the Morpho-Functional Abnormalities Observed in LR-MDS BMSCs

**Figure 2:**
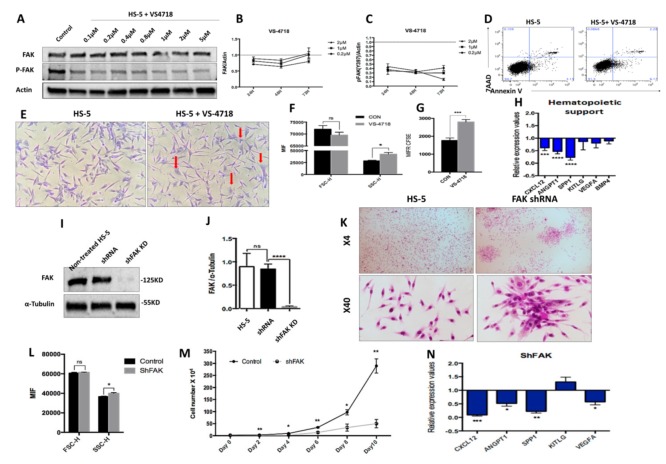
The pharmacological inhibition of Y397-FAK autophosphorylation and short hairpin RNA (shRNA)-mediated knockdown of FAK in HS-5 cells lead to morphological, phenotypic, and functional abnormalities in BMSCs (**A**) Western blot, detection of protein level of FAK and pFAK upon treatment with increasing doses of VS-4718; (**B**,**C**) Relative concentration of FAK and pFAK in HS-5 cells after 24 h, 48 h, and 72 h exposure to increasing doses of VS-4718. (**D**) Viability evaluation of HS-5 cells after exposure to 2 μM VS-4718. (**E**) Representative image of HS-5 cells’ morphology after 72 h exposure to 2 µM VS-4718. Giemsa staining; 200× magnification. Morphological alterations are depicted with red arrows. (**F**) FSC and SSC determination by flow cytometry in HS-5 cells after treatment with 2 µM VS-4718. (**G**) HS-5 cell proliferation assay using carboxyfluorescein-diacetate-succinimidyl-ester (CFSE) tracing (untreated HS-5 cells, black column, n = 3; HS-5 cells exposed to 2 µM VS-4718, grey column, n = 3). (**H**) qRT-PCR measurement of mRNA levels of haematopoiesis-supporting genes after pharmacological inhibition of FAK phosphorylation in HS-5 cells (n_HS-5+VS-4718_ = 3; n_HS-5_ = 3). (**I**,**J**) Western blot, detection of protein level of FAK in HS-5 cells after FAK silencing by shRNA (HS-5, non-infected HS-5 cells, n = 3; shRNA, control shRNA, n = 3; shFAK KD, specific shRNA, n = 3). (**K**) Representative microscopic images at 40×, 100× magnification from HS-5 cultures with (right) and without FAK shRNA knockdown (left), Giemsa staining. (**L**) FSC and SSC evaluation by flow cytometry of HS-5 cells after FAK silencing (Control, control shRNA, n = 3; shFAK, specific shRNA, n = 3). (**M**) Cell growth curves of HS-5 cells after shRNA FAK KD (n = 3) compared to control shRNA (n = 3). (**N**) qRT-PCR measurement of mRNA levels of haematopoiesis-supporting genes in HS-5 cells after FAK silencing by shRNA (n_shFAK KD HS-5_ = 3, n_shRNA control_ = 3). *p* < 0.05(*); *p* < 0.01(**); *p* < 0.001(***); *p* < 0.0001(****).

We sought to determine whether the intrinsic deficiencies of LR-MDS BMSCs were related to the abnormal expression of FAK in stromal cells. Therefore, we evaluated the consequences of FAK inhibition in HS-5 cells, a human stromal cell line derived from a BM healthy donor and immortalised by transduction with the human papillomavirus HPV-16 E6/E7. We used two different strategies to dissect the requirement of FAK within the stromal compartment. We first treated HS-5 cells with the VS-4718 molecule, a selective tyrosine kinase inhibitor that targets the ATP-binding region of FAK (Figure 2A–H).

After treatment of the HS-5 cell line with 2 μM VS-4718 inhibitor, we observed an efficient inhibition of FAK Tyr397 phosphorylation, while the total FAK was mildly decreased (Figure 2A). Interestingly, the inhibition of Tyr397-FAK phosphorylation in HS-5 cells recapitulated the morphologic and phenotypic abnormalities that we previously observed in BMSCs derived from LR-MDS patients (Figure 2E,F). Moreover, the cells displayed a reduced proliferative potential in vitro, as revealed in CFSE-based assays (Figure 2G). In addition, after exposure to the VS-4718 inhibitor, we noticed a significant downregulation of the osteopontin SPP1 gene (encoding for secreted phosphoprotein 1) that controls the osteoblast lineage cells’ growth and differentiation (Figure 2H). Moreover, a significant downregulation of several haematopoiesis-supporting genes, such as CXCL12 (*P* < 0.001) and ANGPT1 (*P* < 0.0001), was detected (Figure 2H).

To study the effect of prolonged FAK inhibition in BMSCs, we performed a stable knock-down of FAK expression using an shRNA strategy. We obtained a reproducible ex vivo study system for FAK-induced BMSC abnormalities, both at the morphological level (Figure 2K,L) and at the functional level, with a decreased proliferative rate (Figure 2M) as well as a significant downregulation of several haematopoietic-supporting genes, including SPP1 (*P* < 0.01), CXCL12 (*P* < 0.001), ANGPT1 (*P* < 0.05), and VEGFA (*P* < 0.05; Figure 2N).

Thus, these data demonstrate that FAK deficiency in BMSCs impairs cell homeostasis and leads to an incapacity to provide any haematopoietic support.

### 3.3. Focal Adhesion Kinase (FAK)-Related Abnormalities in shRNA FAK KD Are Driven by Alterations of Important Signalling Pathways

We next sought to identify the molecular mechanisms triggered by FAK.

**Figure 3:**
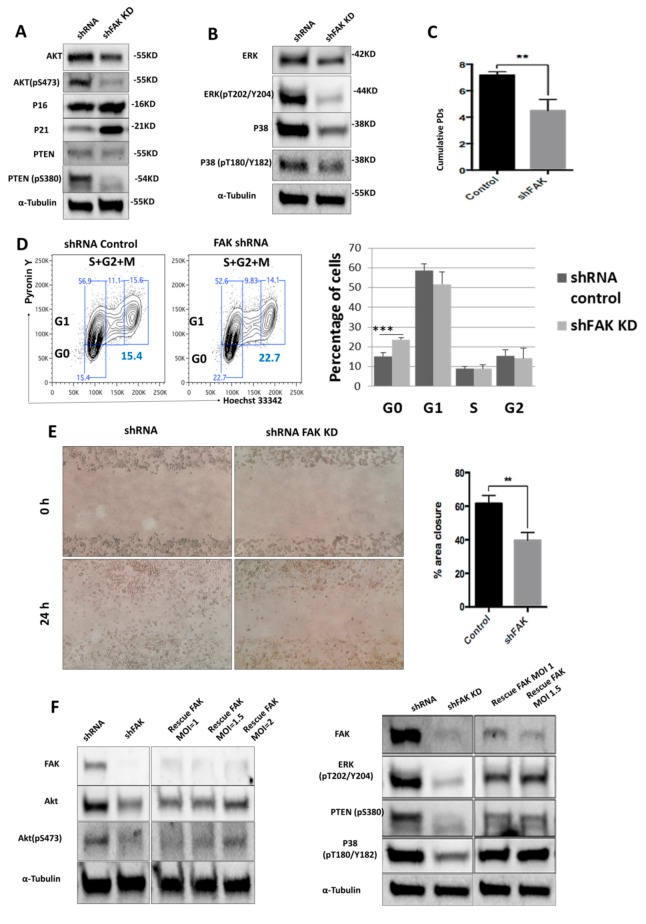
ShRNA-mediated FAK knockdown impairs HS-5 homeostasis by controlling the phosphorylation of several proteins from PTEN-Akt-p21 and ERK-p38 MAPK signalling pathways (**A**,**B**); Western blot analysis of key signalling pathways. (**C**) Doubling time assay of control shRNA and FAK shRNA in HS-5 cells. Initial inoculum cell concentration was 10^5^ cells/cm². (**D**) Cell cycle analysis with Hoechst 33342 and Pyronin Y (n_control shRNA_ = 3, n_shFAK_ = 3). (**E**) Scratch-wound assay of HS-5 cells after FAK shRNA compared to control shRNA (n_control shRNA_ = 3, n_shFAK_ = 3). (**F**) Re-expression of WT FAK in shRNA FAK cells and Western blot analysis of signalling proteins. *p* < 0.01(**); *p* < 0.001(***).

To do so, we used Western blot to evaluate the expression level of several signalling proteins that are known to control the stromal cells’ morphology, proliferation, and differentiation towards osteoblast lineage. Remarkably, we noticed a significant downregulation of Akt (0.61 ± 0.14 for FAK shRNA cells versus 0.96 ± 0.03 for control shRNA, *P* < 0.01) and PTEN (0.36 ± 0.11 for FAK shRNA cells versus 0.74 ± 0.25 for control shRNA, *P* < 0.05), which was associated with a reduced activation of p-Akt (0.21 ± 0.22 for FAK shRNA cells versus 0.83 ± 0.21 for control shRNA, *P* < 0.01), p-PTEN (0.22 ± 0.08 for FAK shRNA cells versus 0.63 ± 0.11 for control shRNA, *P* < 0.01), p-ERK (0.34 ± 0.06 for FAK shRNA cells versus 0.86 ± 0.20 for control shRNA, *P* < 0.01), and p-P38 (0.30 ± 0.13 for FAK shRNA cells versus 0.60 ± 0.13 for control shRNA, *P* < 0.01). Conversely, the cyclin-dependent kinase inhibitor p21 was up-regulated (1.15 ± 0.37 for FAK shRNA cells versus 0.48 ± 0.15 for control shRNA, *P* < 0.001) in shFAK HS-5 cells compared to control shRNA cells (Figure 3A,B). Further, our results showed a significant decrease of cumulative population doubling in FAK shRNA HS-5 cells (4.32 ± 1.72 for FAK shRNA cells versus 7.05 ± 0.35 for control shRNA, *P* < 0.01; Figure 3C) that was correlated with a cell arrest in the G_0_ phase (Pyronin Y^-^ Hoechst 33342^−^, 23.7% ± 1.67% for FAK shRNA versus 15.3% ± 0.90% for control shRNA; Figure 3D).

In addition, we investigated the impact of FAK knockdown in HS-5 in cell migration (Figure 3E). The average percentage of area reduction of wound closure was 39.4% ± 2.54% in FAK shRNA cells versus 60.1% ± 1.76% in control shRNA as calculated in ImageJ 1.51s software.

The partial rescue of FAK expression after re-expression of WT FAK in FAK shRNA cells restored the cell phenotype at the molecular level and led to the normal phosphorylation of all signalling proteins altered by FAK KD (pAkt, pPTEN, p-P38, p-ERK; Figure 3F).

Together, these results suggest that FAK expression is required for the activation of several signalling proteins, which in turn control BMSCs’ homeostasis.

### 3.4. Focal Adhesion Kinase (FAK) Deficiency in BMSCs Triggers the Expansion of Immature Haematopoietic Stem Precursor Cells (HSPCs) and Impairs Their Differentiation towards Erythroid Lineage in Short-Term Co-Culture Conditions

**Figure 4:**
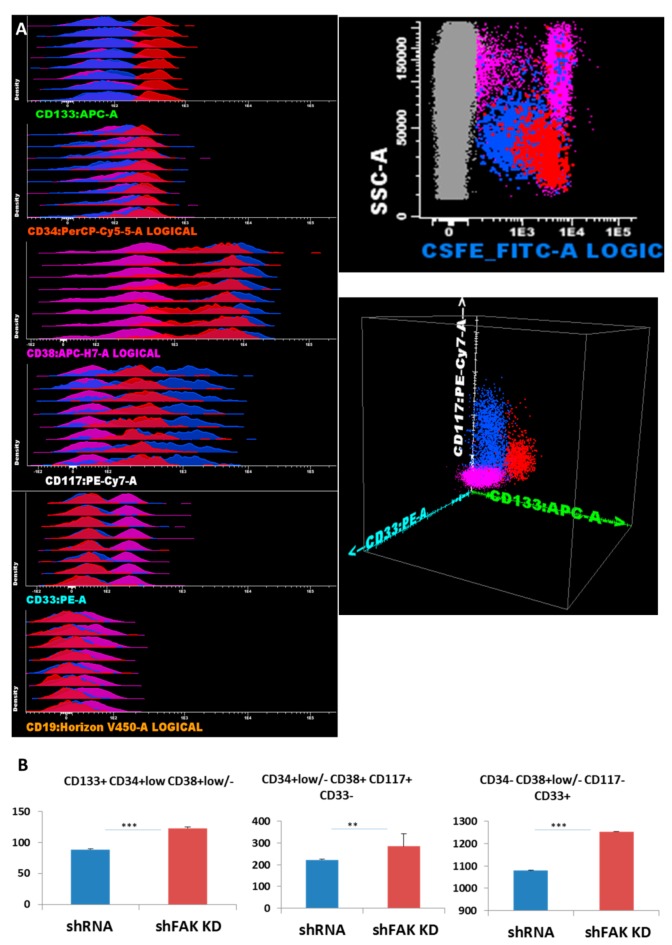
FAK deficiency in HS-5 cells promotes the proliferation of CD34+ HD-HSPCs cells in short-term co-cultures (**A**) Reliable discrimination between the HS-5 cells (grey dots) and CFSE-labelled CD34+ HD-HSPCs (red, blue, and pink dots). Evaluation of HSPCs’ compartment using immature stem cells markers and differentiation antigens. Three main populations are observed: The immature HSPCs CD133+ CD34+low CD38+var CD117+low CD33- CD19- (red dots) and more mature HSPCs CD133- CD34- can be divided by CD38 into two separate subpopulations: CD117+ CD38+ CD33- CD19- (blue dots) and CD38- CD117- CD33+ CD19- (pink dots). (**B**) Absolute cell count of HD-HSPCs recovered after five days of co-cultures between CD34+ HD-HSCs and HS-5 cells (shFAK cells or control shRNA; n_Control shRNA_ = 3, n_shFAK_ = 3). *p* < 0.01(**); *p* < 0.001(***).

Next, we studied whether the deficiency of FAK in HS-5 cells affects the proliferation and differentiation potential of CD34+ HD-HSCs in direct short-term co-cultures. Freshly isolated CD34+ HD-HSCs were labelled with CFSE and incubated on FAK shRNA HS-5. After five days, adherent cells were detached by trypsinisation and evaluated by flow cytometry. Therefore, inside the CSFE+ haematopoietic cells, the HSPC subpopulations were identified using differential expression for immature and more mature immunophenotypic markers, such as CD133, CD34, CD38, CD117, CD19, and CD33 (Figure 4A).

The absolute cell count revealed an overall increase in HSPCs recovered from CD34+ HD-HSC when co-cultured with FAK-deficient HS-5 cells, compared to co-cultures with control shRNA cells (123.3 ± 1.87 HSC/μL versus 88.8 ± 1.23 HSC/μL, *P* < 0.001 for immature HSC CD133+ CD34+low CD38+low/-CD117+low CD19- CD33- cells; 284.5 ± 58.71 HPC/μL versus 223.0 ± 3.86 HPC/μL, *P* < 0.01 for CD133- CD34-/+low CD38+ CD117+hi CD33+ CD19- HPCs; and 1253.8 ± 3.40 HPC/μL versus 1079.5 ± 4.95 HPC/μL, *P* < 0.001 for more mature CD34- CD38+low/- CD117- CD33+ cells; Figure 4B).

**Figure 5:**
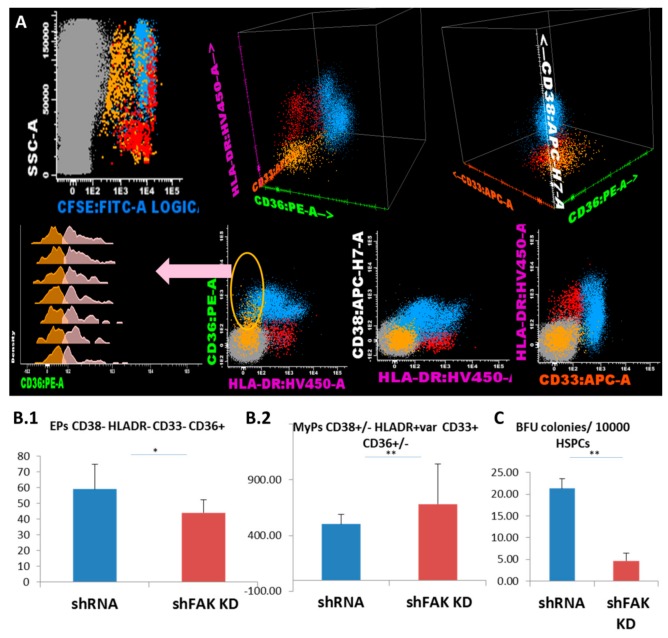
FAK-deficient HS-5 cells negatively regulate the differentiation capacity of CD34+ HD-HSPCs towards erythroid lineage and promote the differentiation of other myeloid progenitors. (**A**) Flow cytometry evaluation of the differentiation capacity of CD34+ HD-HSCs after direct contact with FAK shRNA HS-5 cells during five days versus co-cultures with control shRNA. (**B**) Megakaryocyte/erythroid progenitors (MEPs) CD38- HLADR- CD33- (yellow dots), erythroid precursors (EPs) CD36+ CD33- HLADR- (pale pink dots), and other myeloid precursors (MyPs) CD33+ can be divided by the expression of CD36 in the monocyte-committed precursors CD38+ CD36+ CD33+ HLADR+ (MPs, pale blue dots) and non-monocytic precursors CD38+low/- CD36- CD33+low/- HLADR+ (red dots). The absolute cell count of EPs (n_control shRNA_ = 3, n_shFAK_ = 3; Figure 5B1) and of other MyPs (n_control shRNA_ = 3, n_shFAK_ = 3; Figure 5B2) recovered from CD34+ HD-HSCs from after five days of direct co-cultures with FAK shRNA HS-5 cells compared to control shRNA. (**C**) BFU-E colonies recovered from non-adherent HSPCs after five days of co-cultures with FAK shRNA HS-5 cells compared to control shRNA (n_control shRNA_ = 3, n_shFAK_ = 3). *p* < 0.05(*); *p* < 0.01(**).

We further evaluated the lineage-committed progenitors/precursors inside the HSPC pool based on their expression for CD36, CD33 and HLA-DR markers (Figure 5A). Flow cytometric analysis showed that the proportion and absolute cell count of the erythroid-committed precursors CD34- CD38- CD36+ CD33- HLA-DR low/- obtained from CD34+ HD-HSCs was significantly lower after direct contact with FAK shRNA HS-5 cells compared to control shRNA (44.0 ± 6.0 EPCs/μL versus 59.0 ± 14.0 EPCs/μL, *P* < 0.05; Figure 5B1). Other myeloid precursors including monocyte-committed CD38+/- HLADR+ CD36+ CD33+ cells were significantly elevated (683.0 ± 360.0 MyPs/μL versus 504.7 ± 85.11 MyPs/μL, *P* < 0.01; Figure 5B2). In addition, the ability of HSPCs recovered from co-cultures supernatant to form Burst-Forming Unit-Erythroid (BFU-E) colonies was significantly reduced after contact with FAK shRNA HS-5 compared to control shRNA (4.7 ± 2.50 BFU-E/10^5^ HSPCs versus 21.3 ± 3.66 BFU-E/10^5^ HSPCs; *P* < 0.01; Figure 5B3). 

### 3.5. Haematopoietic Defects Detected in Long-Term Co-Cultures of CD34+ HD-HSCs with Focal Adhesion Kinase (FAK)-Deficient HS-5 Cells Are Driven by Close Contact

**Figure 6:**
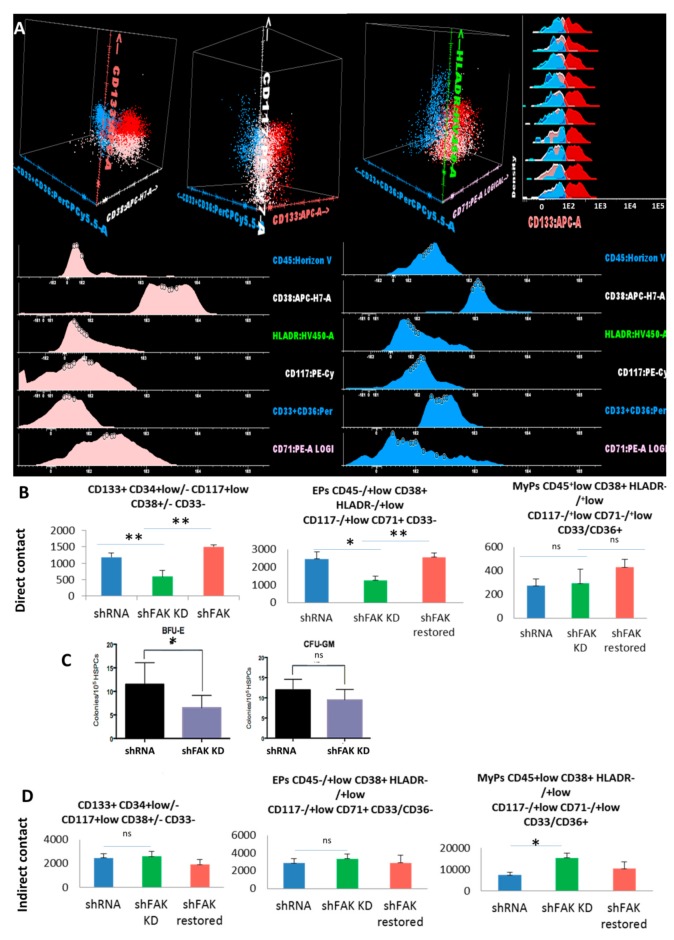
Reduced immature haematopoietic stem cells’ recovery and impairment of erythroid differentiation are observed after co-culture between CD34+ HD-HSCs with FAK shRNA HS-5 cells compared to control shRNA cells. (**A**) Three populations can be identified inside the CSFE+ HSPC population recovered after two weeks of co-culture with FAK shRNA HS-5 or control shRNA: Immature HSCs CD45+low CD133+ HLADR-/+low CD117+low CD71+low CD33/CD36- cells (red dots), erythroid precursors’ EPs CD45-/+low CD133- HLADR-/+low CD117-/+low CD38+ CD33/36- CD71+low (pale pink dots), and other myeloid precursors’ MyPs CD45+low CD38+ HLADR+low CD117-/+low CD33/CD36+ CD71- (blue dots). (**B**) Absolute cell count evaluation of the immature CD133+ HSCs, Eps, and MyPs after two weeks of direct contact with FAK shRNA HS-5 cells compared to control shRNA and FAK shRNA cells after WT FAK re-expression (n_shFAK KD_ = 5, n_shRNA_ = 5, n_shFAK restored_ = 3). (**C**) Burst-forming unit-erythroid evaluation of non-adherent HSPCs recovered from long-term direct co-culture with FAK shRNAHS-5 cells compared to control shRNA. (**D**) Evaluation of the proliferation capacity and erythroid differentiation ability of HSPCs recovered from indirect long-term co-culture between CD34+ HD-HSCs and FAK shRNA HS-5 cells compared to control shRNA and FAK shRNA cells after WT FAK re-expression (n_shRNA Control_ = 5, n_shFAK KD_ = 5, n_shFAK restored_ = 3). *p* < 0.05(*); *p* < 0.01(**).

We also evaluated the differentiation potential of CD34+ HD-HSCs after two weeks of co-cultures with FAK shRNA HS-5 cells (Figure 6). The absolute cell count of the immature CD133+ CD34+low/- CD117+low CD38+/- CD33- cells recovered from adherent fraction after long-term co-cultures between CD34+ HD-HSCs with FAK-deficient HS-5 cells revealed a significant decline compared to those recovered after co-cultures with control shRNA (592.0 ± 188.30 cells /μL versus 1184 ± 134.37 cells/ μL, *P* < 0.01).

In addition, we observed a significant reduction of the CD45-/+low CD38+ HLADR-/+low CD117-/+low CD71+ CD33- erythroid-committed cells in the FAK KD co-culture condition compared to control shRNA (1252.0 ± 239.36 EPCs /μL in direct co-cultures of CD34+ HD-HSCs with FAK-deficient HS-5 cells versus 2460.0 ± 390.65 EPCs /μL in direct co-cultures of CD34+ HD-HSCs with control shRNA, *P* < 0.05; Figure 6B).

In a rescue experiment, the absolute cell count of immature CD133+ HSCs and the EPs-committed cells after two weeks of co-cultures between the CD34+ HD-HSCs and FAK shRNA HS-5 cells that re-express WT FAK were restored and reached levels similar to those detected in long-term co-cultures between CD34+ HD-HSC and control shRNA cells (*P* < 0.01; Figure 6B).

The decreased clonogenic capacity of HSPCs recovered from the supernatant of co-cultures of CD34+ HD-HSCs with FAK shRNA HS-5 cells compared to control shRNA was further confirmed (6.7 ± 3.75 BFU-E/10^5^ seeded HSPC versus 12.3 ± 6.35 BFU-E/10^5^ seeded HSPC, *P* < 0.05; Figure 6C).

The proportion and the absolute cell count of immature HSCs and of committed-HSPCs recovered after long-term indirect co-cultures of CD34+ HD-derived HSCs with FAK shRNA HS-5 cells did not differ from those recovered after indirect co-cultures with control shRNA (Figure 6D).

These data demonstrate that the HSPCs’ homeostasis and differentiation rely on the haematopoietic support provided by direct contact with BMSCs rather than the production of stromal-derived soluble factors. It is thus tempting to speculate that FAK is a key mediator in this direct cell-to-cell communication.

### 3.6. ICAM-1 Drop on FAK KD HS-5 Cells Positively Correlates with LFA-1 and CD44 Decline on HD-Derived HSPCs in Direct Co-Culture

Next, we sought to identify which adhesion molecules were involved in these abnormal adhesion-related processes between FAK KD HS-5 cells and CD34+ HD-derived HSPCs. 

**Figure 7:**
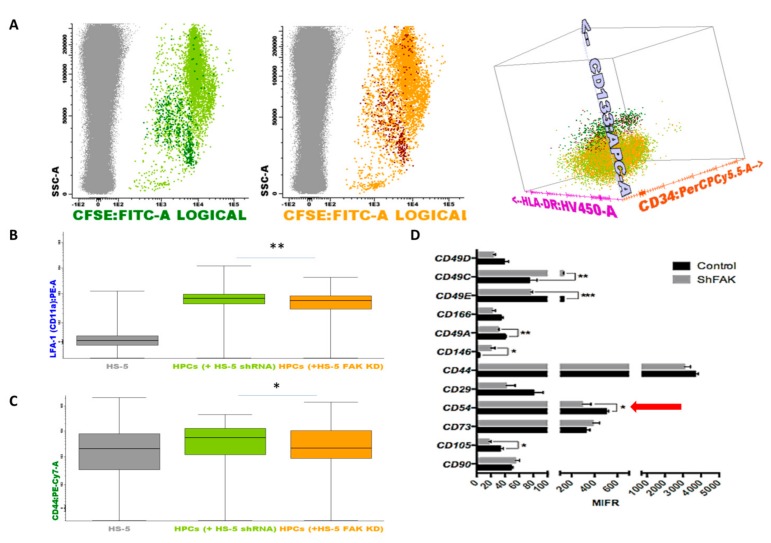
Upon FAK downregulation, the adhesion molecules profile is remodelled in HSPCs and stromal cells when co-cultured in direct contact. (**A**) HSPCs are discriminated from HS-5 cells based on their expression for CSFE (grey dots, non-haematopoietic HS-5 cells; dark green dots, CD133+ HSCs after direct contact with control shRNA cells; light green dots, more mature haematopoietic precursors after direct contact with control shRNA cells Figure 7A left; dark red dots, CD133+ HSCs after direct contact with FAK shRNA HS-5 cells; orange dots, more mature haematopoietic precursors after direct contact with FAK shRNA HS-5 cells Figure 7A middle). Discrimination of HSPC sub-populations based on the expression of CD133, CD34, and HLA-DR markers (Figure 7A right). (**B**,**C**) MFI values of LFA-1 (CD11a) and CD44 on more mature haematopoietic progenitor cells (grey, non-haematopoietic HS-5 cells; light green, HSPCs after direct contact with control shRNA cells; orange, HSPCs after direct contact with FAK shRNA HS-5 cells). Box plots represent the expression level of LFA-1(CD11a) and CD44 (n_shRNA Control_ = 3, n_HSPC+shRNA Control cells_ = 3, n_HSPCs+shFAK KD cells_ = 3). Data were analysed in a merged file composed of three fcs files from HSPCs + shRNA control HS-5 cell co-cultures and three fcs files from HSPCs + shRNA FAK KD HS-5 cell co-cultures. The upper and lower hinges of the box indicate the 75^th^ and 25^th^ percentiles of the data set. The median line within the box represents the median value of the intensity of expression. The whiskers indicate the minimum and maximum data values. (**D**) MFI values of several adhesion molecules evaluated on FAK shRNA HS-5 cells by flow cytometry. *p* < 0.05(*); *p* < 0.01(**); *p* < 0.001(***).

To this end, we performed a flow cytometry screening of the adhesion molecules’ profile of HSPCs collected after two weeks of direct co-cultures (Figure 7). Inside the CSFE+ HSPCs pool, we could discriminate the immature CD133+ HSCs and the more mature CD133- CD34+low/- HSPCs (Figure 7A). We detected a significant downregulation of the expression of lymphocyte function-associated antigen 1 (LFA-1, Figure 7B). The median fluorescence intensity (MFI) values of LFA-1 on HSPCs recovered from CD34+ HD-HSCs co-cultures with FAK shRNA HS-5 cells were 536.5 ± 36.25 versus 694.1 ± 21.74 LFA-1 MFI for the HSPCs recovered from co-cultures with control shRNA cells (*P* < 0.01). A significant reduced expression was also detected for CD44 on HSPCs recovered from co-cultures with FAK KD compared to co-cultures with control shRNA cells (1967.8 ± 446.03 versus 5205.9 ± 676.1 CD44 MFI, *P* < 0.05; Figure 7C).

Similarly, we noticed the downregulation of several integrins including CD49c, CD49e, CD49a, the cell surface glycoprotein MUC18 marker (CD146), and CD105 endoglin in FAK-deficient HS-5 cells. Most importantly, the expression of the ICAM-1 (CD54) molecule, which has a known function in HSCs’ proliferation, was significantly reduced in FAK shRNA cells compared to control shRNA cells (*P* < 0.05; Figure 7D).

These results indicate that FAK-deficient stroma fails to provide adhesion molecules that are needed for the proper functionality of HSPCs.

## 4. Discussion

In our previous work, we showed that BMSCs isolated from patients with MDS displayed an abnormal expression of FAK [17,18].

FAK is a cytoplasmic Tyr kinase with a role in the transduction of integrin signals and plays a key function in controlling cell homeostasis [25]. In solid tumours, it has been demonstrated that FAK is involved in the tumorigenic process by promoting cell migration, invasion, metastasis, and angio- and lymphangiogenesis (reviewed in [21,26]) by controlling apoptosis [27,28] and the self-renewal potential of cancer stem cells [29,30].

Nevertheless, the role of FAK in the pathogenesis of hematologic diseases has received less scholarly attention and remains unknown. Therefore, the contribution of FAK in the haematopoietic niche modelling merits exploration, especially in pathological conditions. In the human leukemic cell line HL-60, the overexpression of FAK is apparently linked to the activation of the PI3K-AKT pathway, and it promotes the expression of NF-κB-mediated inhibitor-of-apoptosis proteins (IAPs), which in turn warrant apoptotic inhibition by blocking the caspase-3 cascade [31]. Only one study mentions the fact that FAK+ AML-derived BMSCs have a decreased clonogenic activity due to an increased senescence [32], but there is no direct evidence showing that FAK abnormal expression in BMSCs contributes to leukaemia development and by which mechanism.

In this context, we sought to investigate the consequences of the abnormal expression of FAK in the BM microenvironment in MDS, a pre-leukemic setting.

We observed that FAK-deficient stromal cells and shRNA FAK HS-5 cells failed to support the normal haematopoiesis by several mechanisms. FAK-deficient BMSCs and FAK KD HS-5 cells displayed characteristics of the so-called ‘age state’, such as an abnormal morphology, an impaired proliferation, and a compromised osteogenic differentiation capacity. These abnormal processes might contribute to the incapacity of the BM microenvironment to produce an efficient number of supporting cells for a normal haematopoiesis. The functional abnormalities observed in FAK-deficient BMSCs are likely related to the role of FAK in activating different signalling pathways, such as the PTEN-AKT-p21 axis involved in BMSCs’ proliferation, survival, and differentiation, as well as in the maintenance of normal cell morphology and metabolism [33,34]. The ERK-P38 from the MAPK pathway has a key role in BMSCs’ motility [35]. In support of this hypothesis, we observed that FAK KD in HS-5 cells impairs the expression and activation of several proteins from the PTEN-AKT-p21, ERK, and p38 MAPK signalling pathways. The paradoxical decline of both proteins Akt and PTEN in FAK KD stromal cells could be explained by ablation of p110beta protein (also known as Pik3cb) within a rescue mechanism as observed in PTEN-deficient tumours [36]. The partial re-expression of FAK in FAK shRNA HS-5 cells restored the phosphorylation of several signalling proteins, including PTEN, ERK, and p38.

In addition, FAK-deficient stromal cells selected from LR-MDS and the HS-5 cell line treated with VS-4718 selective FAK inhibitor as well as FAK shRNA HS-5 cells showed a decreased expression of the CD54 (ICAM-1) adhesion molecule. Interestingly, it has been previously demonstrated that ICAM-1 deficiency in the BM niche in ICAM-1-/- mice impaired the quiescence and repopulation of HSCs through an abnormal retention of HSCs in the BM, which is a prerequisite for proper HSC function [15].

We also observed that, after short-term direct contact with FAK shRNA HS-5 cells, CD34+ HD-HSCs showed an increased proliferation, which was equally observed after the neutralisation of ICAM-1 [15]. In our experiments, this initial increased proliferation was followed by the exhaustion of the immature CD133+ CD34+ haematopoietic progenitors in long-term co-cultures. Moreover, the HSPCs’ abnormalities were related to the reduced expression of several haematopoietic-supporting genes in BMSCs from LR-MDS and in FAK shRNA HS-5 cells. Among those genes, we noticed SDF1, also known as C-X-C motif chemokine 12 (CXCL12), Angpt1, and SPP1. This observation is in line with previously reported data, according to which CXCL12 deletion in mesenchymal progenitors using Prx1-cre was associated with a marked loss of HSCs’ long-term repopulating activity, HSC quiescence, and an impairment of the ability to induce multi-lineage reconstitution [37]. Further, the Tie2/Ang-1 signalling pathway is also critical for the maintenance of HSCs in a quiescent state in the BM niche [38].

In addition, we observed an impairment of differentiation towards the erythroid lineage of CD34+HD-HSCs after direct contact with FAK KD HS-5 cells. Notably, these functional and differentiation abnormalities were not detected after indirect co-cultures of CD34+HD-HSCs with FAK KD HS-5 cells. Hence, the molecular mechanism does not seem to be related solely to the alteration of stromal-derived soluble factors, such as cytokines or growth factors, but rather to direct contacts via adhesion molecules.

It has been suggested that the binding of ICAM-1 expressed by stromal cells with αLβ2 (also called LFA-1, or CD11a) on HSCs facilitates their proper retention and homeostasis in the BM [15]. The normal expression of CD44 on HSPCs within the BM microenvironment is also important for the homing and lodgement of adult HSPCs [39]. Among these molecules, we noticed the drop of several integrins, such as CD49c, CD49e, and CD49a; of the cell surface glycoprotein MUC18 marker CD146 (which is associated with proangiogenic [40] and chondrogenic differentiation properties [41]); CD105 endoglin; and the ICAM-1 (CD54) molecule with a role in HSCs’ proliferation control [15].

Our experiments showed a significant diminution of LFA-1 and CD44 expression in CD34+ HD-HSCs after long-term direct co-cultures with FAK KD HS-5 cells.

## 5. Conclusions

Collectively, these data show that impairment of FAK expression or its inactivation in BMSCs is correlated with ICAM-1 deficiencies and less expression of several haematopoiesis-supporting genes, which in turn affects HSCs’ proper retention through abnormal adhesion processes between HSPCs and BMSCs, including LFA-1 and CD44.

These results may lead to the development of a therapeutic strategy to recover FAK expression in BMSCs from LR-MDS patients in order to improve cytopenias, especially anaemia.

## Figures and Tables

**Table 1:** Biological and clinical characteristics of the myelodysplastic syndrome (MDS) patients included in this study.

Sample	Age (y)	WHO 2018	Hb (g/dL)	Plt (×10^9^/L)	ANC (×10^9^/L)	Karyotype	%BM Blasts	IPSS
MDS_1	76	RARS	9.8	269	8.45	46,XY (20)	0.5	Low
MDS_2	73	MDS-5q	8.2	201	4.48	46, XX del(5)(q13q33) (18)/46, XX (12)	3	Low
MDS_3	79	MDS-U	7.8	866	6.71	46,XY (20)	2	Low
MDS_4	76	RCMD	8.9	198	0.8	46,XY (20)	2	Int-1
MDS_5	89	RCMD	7.5	230	2.46	47, XY,+8(22)/45,X,-Y (2)/46, X,-Y,+8 (3)/46,XY (3)	2.5	Int-1
MDS_6	81	RCMD	6.3	125	2.19	46,XY (20)	3.5	Int-1
MDS_7	84	MDS-5q	10.8	235	1.43	46, XY, del(5)(q13q33) (6)/46, XY (14)	2	Low
MDS_8	76	RA	8.8	298	3.92	46, XY (20)	0	Low
MDS_9	85	RCMD	12.3	104	1.57	46, XY (20)	1.5	Int-1
MDS_10	85	RCMD	11.6	162	2.85	46, XY (20)	1.5	Low
MDS_11	77	RA	7.7	346	3.95	46, XX (20)	1.5	Low
MDS_12	84	RAEB-1	10	306	7.76	46, XY (20)	5.5	Int-1
MDS_13	75	RARS	99	260	8.41	46, XY (20)	0	Low
MDS_14	70	RAEB-1	15.1	389	1.46	46, XX (20)	4.5	Low
MDS_15	70	RCMD	8.8	394	2.38	46, XX (20)	2	Low
MDS_16	84	RCMD	12.9	53	1.98	46, XY (20)	4.5	Int-1
MDS_17	89	RCMD	9.2	373	5.02	46, XX (20)	1	Low
MDS_18	88	RA	10.2	94	2.53	46, XY (20)	0	Low/Int-1
MDS_19	87	RCMD	9.3	52	1.39	46, X,-Y,+15 (30)	2.5	Int-1
MDS_20	79	RCMD	7.1	268	1.77	46, XX (20)	3.5	Int-1
MDS_21	77	RCMD	8.4	55	2.08	46, XY, del(13)(q12q14) (17)/46, XY (3)	1	Int-1
MDS_22	84	MDS-5q	10.1	286	5.72	46, XX, del(5)(q13q33) (15)/46, XX (5)	2	Low
MDS_23	77	RA	7.7	346	5.69	46, XX (20)	1.5	Low
MDS_24	79	RAEB-2	7.5	256	13.59	46, XX, del(5)(q13q31) (20)	15	Int-2
MDS_25	72	RAEB-2	5.6	59	0.31	46, XY (20)	12	Int-2
MDS_26	65	RAEB-2	8.9	61	0.16	46, XX, del(5)(q13q33)(1)/48~51,S1,+1,add(3)(q11),+6+11-15,-17,-18,-21,+1~5mar[cp17]/96.sdlx2 (1)/46, xx (1)	17	High
MDS_27	84	RAEB-2	9.4	59	2.05	41~46, X,-X,-3,-5,-6,-15,add(16)(q2?4),-18,+2~7mar[cp19]/46, XX (1)	15	High

Hb, Haemoglobin; Plt, Platelets; ANC, absolute neutrophil count; WHO, World Health Organisation classification of Hematologic Malignancies; IPSS, international prognostic scoring system; MDS-5q, myelodysplastic syndrome with isolated del5q; MDS-U, myelodysplastic syndrome-unclassified; RAEB, refractory anaemia with excess blasts; RCMD, refractory cytopenia with multilineage dysplasia; RA, refractory anaemia; RARS, refractory anaemia with ring sideroblasts.

**Table 2:** List of antibodies for bone marrow stromal cells (BMSC) discrimination and immunophenotype characterisation by flow cytometry.

*Antibodies*	*Vendor*	*Clone*	*Fluorochrome*	*Concentration*
**Mouse anti-CD90**	BD Biosciences	5E10	FITC	5 μg/100 μL
**Mouse anti-CD73**	BD Biosciences	AD2	APC	5 μg/100 μL
**Mouse anti-CD105**	BD Biosciences	266	PerCp-Cy5.5	5 μg/100 μL
**Mouse anti-CD146**	BD Biosciences	P1H12	PE-Cy7	5 μg/100 μL
**Mouse anti-CD106**	BD Biosciences	51-10C9	PE	20 μg/100 μL
**Mouse anti-CD54**	BD Biosciences	VI A095	PE	20 μg/100 μL
**Mouse anti-CD44**	BD Biosciences	VI A092	PE-Cy7	2 μg/100 μL
**Mouse anti-CD49a**	BioLegend	TS2/7	PE-Cy7	2 μg/100 μL
**Mouse anti-CD49c**	BD Biosciences	C3II.1	BV421	2 μg/100 μL
**Mouse anti-CD49d**	BD Biosciences	9F10	APC-H7	5 μg/100 μL
**Mouse anti-CD49e**	BD Biosciences	IIA1	BV421	5 μg/100 μL
**Mouse anti-CD166**	BD Biosciences	3A6	PE-Cy7	5 μg/100 μL
**Mouse anti-CD29**	BD Biosciences	VI A093	PE	5 μg/100 μL
**Mouse anti-CD45**	BD Biosciences	HI30	V500	5 μg/100 μL

**Table 3:** Real-time PCR primer sequences.

Gene	Forward Primer (5′-3′)	Reverse Primer (5′-3′)
***ANGPT1***	AGCGCCGAAGTCCAGAAAAC	TACTCTCACGACAGTTGCCAT
***KITLG***	AATCCTCTCGTCAAAACTGAAGG	CCATCTCGCTTATCCAACAATGA
***SPP1***	GAAGTTTCGCAGACCTGACAT	GTATGCACCATTCAACTCCTCG
***CXCL12***	ATGAACG CCAAGGTCGTG	ACATGGCTTTCGAAGAATCG
***VEGFA***	CTACCTCCACCATGCCAAGT	GCAGTAGCTGCGCTGATAGA
***GAPDH***	AATCCCATCACCATCTTCCAGG	AGAGGCAGGGATGATGTTCTGG
PTK2*	CCAAATGGAGCCAGTGAACCT	AAGCACGTGGCCTGCTATG

* human PTK2 gene encoding FAK.

**Table 4:** List of antibodies for haematopoietic stem precursor cells (HSPCs) immunophenotyping.

*Antibodies*	*Vendor*	*Clone*	*Fluorochrome*	*Concentration*
**Mouse anti-CD33**	BD Biosciences	P67.6	APC	10 μg/100 μL
**Mouse anti-CD34**	BD Biosciences	8G12	PerCp-Cy5.5	5 μg/100 μL
**Mouse anti-CD38**	BD Biosciences	HB7	APC-H7	5 μg/100 μL
**Mouse anti-HLA-DR**	BD Biosciences	L243	V450	5 μg/100 μL
**Mouse anti-CD71**	BD Biosciences	C2	PE	20 μg/100 μL
**Mouse anti-CD117**	BD Biosciences	104D2	PE-Cy7	5 μg/100 μL
**Mouse anti-CD33**	BD Biosciences	P67.6	PerCp-Cy5.5	10 μg/100 μL
**Mouse anti-CD133**	Miltenyi Biotec	REA753	APC	5 μg/100 μL
